# Acknowledgment to the Reviewers of *Children* in 2022

**DOI:** 10.3390/children10020174

**Published:** 2023-01-17

**Authors:** Children Editorial Office

**Affiliations:** MDPI AG, St. Alban-Anlage 66, 4052 Basel, Switzerland

High-quality academic publishing is built on rigorous peer review. *Children* was able to uphold its high standards for published papers due to the outstanding efforts of our reviewers. Thanks to the efforts of our reviewers in 2022, the median time to first decision was 18 days and the median time to publication was 40 days. Regardless of whether the articles they examined were ultimately published, the editors would like to express their appreciation and thank the following reviewers for the time and dedication that they have shown *Children*:
Abate, AndreaLibes, JaimeAbbasi, YaseenLičková, MartinaAbdalla, ElsayedLieber, JustusAbdallah, Atiyeh M.Lieberman, LaurenAbdović, SlavenLier, HeikoAbdul Aziz, Dayang AnitaLiley, HelenAbdullah, RozainiLilienthal, IngridAbdulrahman Jairoun, AmmarLim, Choon GuanAbid, GhulamLim, JongbeomAbitbol, Carolyn L.Lim, LyndonAbou-Rizk, JoanaLim, Mathew Albert Wei TingAbramowicz, PawełLima, RitaAbrams, StephenLin, Chien-HengAbu-Awwad, AhmedLin, Esther Ching LanAbuzerr, SamerLin, JessicaAccadbled, FranckLin, Jiuann-Huey IvyAceti, AriannaLin, Tsung LiAchiron, AsafLin, Wei-ShengAchmad, HarunLin, Yu-ChengAdam, RaziaLin, Yuh‐YihAdamiak, ZbigniewLindner, ThomasAddison, CliftonLindsay, PietruszewskiAdeleke, Oluwatoyin A.Lines, LaurenAdeyinka, AdebayoLinhares, Maria BeatrizAdla, TheodorLinnér, AgnesAdouni, MalekLipiński, PatrykAdrada-Rafael, SergioLipowicz, AnnaAdrian, LunguLirio Romero, CristinaAdriawan, Ignatius RyanLister, Rolanda L.Adsuar Sala, José CarmeloLitwinska, MagdalenaAedma, Kapil KiranLiu, Chin-HsuanAfolabi, AfolawemiLiu, Chin-TingAfra, PegahLiu, HairuiAfshari, ArashLiu, Sze YanAgati, SalvatoreLiu, XiaoqingAgha, Hala MounirLiu, ZhongweiAghlmandi, SoheilaLivzan, Maria A.Agrawal, VikashLizandra, JorgeAgresta, FerdinandoLizano-Díez, IreneAguaded-Ramírez, EvaLlamas-Ramos, InésAgudelo Velásquez, Carlos AlbertoLlaurado, MireiaAgudelo-Suárez, Andrés AlonsoLlopis-González, AgustínAhmed, NaseerLlorente, Carlos MartinAhmedien, DiaaLo Giudice, RobertoAhn, Jeong-AhLo, ChristopherAiroldi, GiorgioLobo, MicheleAkangire, GangaramLockridge, OksanaAkbari, MitraLoertscher, MartinAkelma, ZulfikarLogan, Nicole E.Akintayo, ThomasLoghmanee, DariusAkombi-Inyang, Blessing J.Loh, Amos H. P.Akota, IlzeLoi, EleonoraAksenov, Daniil P.Lois, BeckyAl Omari, OmarLöllgen, RuthAlamri, MajedLombamo, Getahun ErsinoAlatorre-Jimenez, Moises AlejandroLombardi, LuciaAlbert, Gregory W.Lonardo, FortunatoAlcaide, MartaLond, BarbaraAlcaraz-Estrada, Sofía LizethLong, YichengAldoory, LindaŁoniewska, BeataAlejandra, Medina-RiveraLopes, SílviaAlexandropoulou, IoannaLópez Escribano, CarmenAlhussain, MahaLópez, MiguelAli, Ahmed S.Lopez-Agudo, Luis AlejandroAli, AmanatLópez-García, Juan CarlosAlizadeh, RezaLópez-García, SergioAl-Khalifa, KhalifaLópez-Gil, José FranciscoAllegaert, KarelLópez-Izquierdo, InmaculadaAlloghani, MohamedLoreti, ClaudiaAlMaghaslah, DaliaLoron, GauthierAl-Marzooq, Farah IbrahimLosada Puente, LuisaAlmehmadi, MazenLoscocco, Giuseppe G.Almeida, Ana SusanaLoster, Jolanta E.Almeida, Carlos Manuel TorresLosurdo, GiuseppeAlmeida, JoannaLotfollahzadeh, SaranAlmeida, TelmaLouder, TalinAlmeida-Neto, Paulo Francisco DeLouie, JessicaAlmendra-Pegueros, RafaelLouie, LoboAlMuneef, MahaLovett, MarlinaAlonso-Arroyo, VeronicaLovett, Marlina E.Alqraini, FaislLovrecic, MercedesAl-Rashaida, MohammadLovrenski, JovanAlriksson-Schmidt, AnnLovvorn, Harold N.Alshaikh, Belal N.Lowrie, LiaAlshammary, DeemaLoyola-Rodríguez, Juan PabloAlsowaida, Yazed SalehLu, JinjinAl-Talib, TanyaŁubiech, KatarzynaAltuna, MirenLubrano, RiccardoÁlvarez Muelas, AnaLucchese, AlessandraAlvarez, Ofelia A.Luciani, MichelangeloAlzawad, ZainabLucke-Wold, BrandonAmadori, FrancescaLudovichetti, Francesco SaverioAmatria, MarioLuglietto, DavideAmbili Vijayakumari, AnupaLukas, MilanAmbrona-Zafra, DavidŁukasik, JoannaAmend, StefanieLundqvist, ChristoferAmezcua-Aguilar, TeresaLungaro, LisaAmin, ZubairLuque De La Rosa, AntonioAmirav, IsraelLuque-Vara, TrinidadAmo, Rocío OrtizLvovschi, Virginie ÈveAmornyotin, SomchaiLynch, Jennifer M.Amudhan, SenthilLystad, Reidar P.Amzar, DanielaM. Chisthi, MeerAnagnostopoulos, IoannisMa, JianpingAnand, SachitMa, TongcuiAnastasio, Albert ThomasMaase, Rose E.Anders, Jennifer F.Mabasa, LawrenceAndersen, SusanMacambira, Simone GarciaAnderson, AlisonMacca, LauraAnderson, Sarah ElizabethMaccagnano, GiuseppeAndréasson, StenMacchiaiolo, MarinaAndreu-Pejó, LauraMacDowell, PaulaAndrews, SinéadMachairas, NikolaosAndriessen, KarlMack, IsabelleAndrievskaya, IrinaMack, Joana M.Andronesi, AndreeaMacLeod, StuartAneji, ChiamakaMacNeill, LeighaAngelopoulou, GeorgiaMaddox, Ryan A.Anghel, LarisaMadeira, GeoffreyAngueyra, ChantalMadinabeitia, IkerAntonakopoulos, NikolaosMadrona, Pedro GilAntonelli, ElisabettaMaeda, TomokiAntonescu, ElisabetaMaekawa, MakiAntonicelli, AlbertoMaepa, Mokoena PatronellaAntoniou, Maria-ChristinaMaestri, FrancescoAntonoaea, PaulaMaestri, LucianoAnwer, RaziqueMagalhães, CátiaApa, EnricoMaguire-Jack, Kathryn L.Aparisi, DavidMahadevaiah, GuruprasadAppenzeller, SimoneMahgerefteh, JosephArakawa, EijiMajer, MarjetaAraki, TakashiMajewska, Katarzyna AnnaArán-Filippetti, VanessaMakarewich, Christopher A.Aranha, ÁgataMakimoto, AtsushiAranha, AnilMakiyan, ZograbAranoff, GeraldMalagón-Rojas, JeadranAraujo, John JairoMalak, RoksanaAravindraja, ChairmanduraiMalatesti, NelaArbildo-Vega, HeberMalushko, ElenaAreti, PanaouraMamen, AsgeirArgueta-Figueroa, LilianaManan, WanArias-Pujol, EulaliaManfredi, MarcoArigliani, MicheleMangweth-Matzek, BarbaraAris-Meijer, JudithMania, AnnaArkachaisri, ThaschaweeManiaci, AntoninoArneitz, ChristophManikowska, FaustynaArnold, Robert W.Mannarino, SavinaArranz, EnriqueManolescu, Diana LuminitaArsoniadis, Gavriil GeorgeMansilla, Juan RodríguezArtopoulou, Ioli IoannaMansour, EliArtym, JolantaMansur, Adel H.Arul, NandiniMantadakis, ElpisAsam, Aiman ElMantilla Herrera, Ana MariaAsare, MattManuel, Solmaz P.Asbrand, JuliaManzano Sánchez, DavidAsim, MuhammadMarano, PietroAstigarraga, ItziarMaras, NevenkaAtzl, Victoria M.Marc, Monica StelutaAu, Sunny Chi LikMarchandin, HélèneAubert, Adrien M.Marchesi, AlessandraAugusto, OrvalhoMarco, Federico DeAura Loredana, PopescuMarenčáková, JitkaAuzenbaha, MadaraMargraf-Stiksrud, JuttaAvasiloaiei, AndreeaMariani, IlariaAyabe, MakotoMarimon, Jose MariaAydin, MalikMarin, JoseAydın, SonayMarin, LucaAyeleke, Reuben OlugbengaMarino, AchilleAyers, MaryMarino, MariaAyusawa, MamoruMarinos, GeorgiosBaba, KenjiMarín-Paz, Antonio-JesúsBabar, GhufranMarković, Maša VukčevićBabicki, MateuszMarkovic, SrdjanBabik, IrynaMarks, Jeremy D.Babinska, KatarínaMarques, Joana MendesBabughirana, GeoffreyMarques, Maria Do CéuBacaro, ValeriaMarques, PatríciaBaceviciene, MigleMarrone, ChiaraBachou, HanifaMarston, Joan M.Badau, AdelaMartín Antón, Luis JorgeBaek, Seung-HakMartin, JoelBaena, SalvadorMartin, Richard H.Bafor, AnirejuoritseMartin, VictorBaglioni, ValentinaMartinelli, VictorBagó, JoanMartínez Fernández, María CristinaBagyinszky, EvaMartínez Galiano, Juan MiguelBairagi, AnjanaMartinez-Nieto, LourdesBajric, ElmedinMartínez-Olmos, Francisco J.Bal, Zümrüt ŞahbudakMartínez-Ramón, Juan PedroBalakathiresan, Nagaraja S.Martínez-Rojano, HugoBalcombe, LukeMartinez-Vázquez, SergioBalcoş, CarinaMartinho, José PedroBale, AlanMartins, Ana TeresaBalice-Bourgois, ColetteMartins, CátiaBall, Hope C.Martins, DianaBalwierz, WalentynaMartins, JoãoBalzanelli, CristianoMarto, Carlos MiguelBambharoliya, TusharMartu, IoanaBanac, SrđanMartynowicz, HelenaBanach, KatarzynaMarzieh, Saei Ghare NazBanasiuk, MarcinMasiran, RuzianaBanerjee, JinetaMaslak, KarolinaBanerjee, KalpitaMasoller, NarcisBang, Kyung-SookMassa, ValentinaBañuelos Marco, BeatrizMassler, UteBaranowski, ThomasMasutani, SatoshiBarański, KamilMatás Castillo, MercedesBarbaro, KatiaMathews, RanjivBarbi, EgidioMathias, MaxwellBarbieri, ElisaMatos, RuiBarbounaki, StavroulaMatsubara, ShigekiBargiel, JakubMatsuda, YuheiBarnett, AnnaMatsumiya, WataruBarqawi, Hiba JawdatMatsuno, Alessandra K.Barragan-Jason, GladysMatsuo, MuneakiBarreto, JessicaMatsuzaki, ShinyaBarrett, Katherine J.Matsuzawa, AkemiBarrett, RustyMattsson, SofiaBarrios Fernández, SabinaMaurotti, SamanthaBarsties Von Latoszek, BenjaminMaya-Enero, SilviaBartkowski, JohnMayta-Tovalino, FrankBartoli, FabioMazur, JoannaBaşal, YeşimMazur, MartaBashore, LisaMazurek, HenrykBašković, MarkoMazurkiewicz, Dariusz WojciechBasnayake, ChamaraMazurkiewicz, DominikaBasrowi, Ray W.Mazza, ElenaBataga, Simona MariaMazza, ElisaBates, Carolyn R.Mazzucato, MonicaBatsalova, TsvetelinaMcAllister, Katherine AnnBattaglia, SimoneMcCarthy, HelenBattista, Valeria Marinella AugustaMcCleery, JulieBauce, BarbaraMcClement, SusanBausela Herreras, EsperanzaMcConkey, RoyBaúto, Ricardo VenturaMcDonald, Fiona E. J.Bauwens, JorgenMcDonell, James R.Bazan-Socha, StanislawaMcEwen, Scott T.Beasley, Gary S.McKay, SamuelBedetti, LucaMcKelvie, Penny A.Begdache, LinaMcMahon, TadghBeghetti, IsadoraMcMillen, Jennifer D.Behzadi, PayamMcmorrow, TaraBeier, MichaelMecías-Calvo, MarcosBeijers, RoserietMeda Lara, Rosa MarthaBelančić, AndrejMedhat, DaliaBelmatoug, NadiaMędrzycka-Dąbrowska, WiolettaBeltzer, ChristianMeesters, KevinBen Ari, AmichaiMegari, KalliopiBenedetti, M. G.Megremis, SpyridonBenedetto, LoredanaMehanna, EmanBenevento, MarcelloMehra, KashishBenna, PaoloMehta, RukshanBentivegna, EnricoMeinzer, AndreasBerbrayer, DavidMelit, Lorena ElenaBerce, VojkoMena-Tudela, DesiréeBerdina, Olga N.Mendes, PedroBerger, NinaMendizabal-Espinosa, Rosa MariaBergeron, MathieuMendoza-Muñoz, MaríaBerhane, Hanna Y.Mendoza-Sagaon, MarioBerki, TamásMenescardi, CristinaBerlanga Macías, CarlosMeo, Ermelinda DeBernad, ElenaMerckaert, Sophie RosaBernasconi, DavideMeregildo Rodriguez, Edinson DanteBernat, John A.Merino-Andres, JavierBernet, WilliamMerquiol, FanetteBerral de la Rosa, Francisco JoseMessina, GabrieleBers, Marina UmaschiMessina, RaffaellaBertamini, GiulioMeštrović, SenkaBerthelot, LaurelineMetrock, Laura K.Bertolotto, AntonioMeyer, BeauBeversdorf, DavidMeynaar, Iwan AugustBeyer, Eric C.Mezawa, HidetoshiBeyer, KatharinaMiccio-Fonseca, L. C.Bhandari, RashmiMichelogiannakis, DimitriosBhat, Ballambhattu VishnuMichihata, NobuakiBhatia, DivyaMičule Fjodorova, IlonaBhatia, HimanshiMiddleton, Kimberly R.Bhattacharjya, SutanukaMidrio, PaolaBhattacharya, SoumeMielmann, AnnchenBhoopalan, SenthilMigliorini, LauraBiagi, LyviaMignon, SylviaBiałek-Dratwa, AgnieszkaMigoń, DorianBianchetti, Mario G.Mihai, MihaelaBianchi, RobertoMihaicuta, StefanBianco, FrancescoMihálik, JaroslavBianco, Maria RitaMihanovic, JakovBıçakcı, ÜnalMihatsch, WalterBici, KejdMikhailov, TheresaBickel, ScottMilanova, AneliyaBidabadi, ElhamMilanovic, FilipBienko, MarekMileder, Lukas P.Bieńkowski, CarloMilewska, MagdalenaBiermann, DanielMiller-Kasprzak, EwaBigoni, MarcoMilone, RobertaBilbault, ClaireMilošević, DankoBilge, HuzeyfeMilostić Srb, AndreaBilgin, EmreMilostić-Srb, AndreaBilińska, Zofia T.Milota, TomasBillich, NatassjaMilovanović, IvanaBin, KimMin, Hyun-JinBindi, EdoardoMin, JinhongBinic, IvaMinchella, Peter A.Biniwale, Manoj A.Minghelli, BeatrizBinos, ParisMinh Duc, NguyenBisaccia, GiandomenicoMinoura, AkiraBisharat, MayMirabelli, MariaBisiacchi, PatriziaMirea, AndradaBizerea-Moga, Teofana-OtiliaMirel, SimonaBlack, MichelleMirmosayyeb, OmidBlackmore, MarieMirzaei, RasoulBlackstone, CraigMišak, Zrinjka MatekBlancas-Galicia, LizbethMishra, DevendraBlanchon, SylvainMiskulin, MajaBlanco, ClarissMisra, SnigdhaBlanco, EstelaMitsea, AnastasiaBlanco, IgnasiMizia-Malarz, AgnieszkaBłażejewski, AndrzejMlinaric, MatejBlohm, Martin E. G.Močnik, MirjamBobescu, ElenaModi, ShikhaBoccatonda, AndreaModica, RobertaBodaghi, DariushModrzejewska, JustynaBoedeker, WolfgangMoga, AndreeaBoettcher, JohannesMohamed, Adel A.Bogdan, NeamtuMohamed, Mohd S.Boggiano, Victoria L.Mohd Rani, Mohd DzulkhairiBohannon, Richard W.Mohiuddin, Mahi M.Boito, SimonaMoisan, FrancoisBoix-Ochoa, JoseMoles, Jean-PierreBolaños, María De LourdesMolina Fernández, Antonio JesúsBolcato, MarilenaMolino, AntonioBoles, Jessika C.Molnár, DénesBolesławska, IzabelaMombarg, RemoBollig, GeorgMomose, HitoshiBonifazi, DonatoMondanelli, NicolaBonneton-Botté, NathalieMondardini, Maria CristinaBonomi, AliceMonroy Torres, RebecaBoolani, AliMonroy-Jaramillo, NancyBoomsma, Martijn F.Montani, GiancarloBora, SamudraguptaMonteiro, AntónioBorovečki, AnaMonteiro, CristinaBorrelli, PaolaMontúfar-Robles, IselaBorzelli, DanieleMoore, AmyBotero-Meneses, Juan SebastiánMooren, TrudyBouchekioua, YoucefMorais, NunoBouffet, EricMoraleda Sepúlveda, EstherBoukhobza, SarraMorales Chaine, SilviaBoukhris, Mohamed RiadhMorales Rodríguez, Francisco ManuelBourguiba, RimMorales, Angelica M.Boursier, GuilaineMorales, Maria AuroraBoutiuc-Kaiser, AlinaMoramarco, StefaniaBovo, RobertoMorasiewicz, PiotrBoyden, JackieMorawska-Kochman, MonikaBraga, MiriamMordenti, MarinaBrajković, LovorkaMoreau, AlainBrancato, AnnaMorel, Fanny B.Branco, FranciscoMoreno, Déborah SanabriasBrandon, ThomasMoreno-Álvarez, AnaBraun, TinaMoretto, EnricoBrawura-Biskupski-Samaha, RobertMorgan, HaniBreitkopf, RobertMorgan, IanBrena, MichelaMorgan, Kyle J.Brilli, RichardMorita, NoriteruBringas, Maria LuisaMorosan, HaricleaBrisca, GiacomoMorrone, PietroBrito, RitaMortensen, Kristian H.Brkic, Faris F.Moscatelli, FiorenzoBrniak, WitoldMoschino, LauraBroadbent, JonathanMoses, JonathanBrodosi, LuciaMosler, DariuszBroman, Lars MMoszak, MałgorzataBronikowska, MałgorzataMoszyńska, MałgorzataBronikowski, MichałMotamed, CyrusBröning, SonjaMotch Perrine, SusanBrookman, RuthMotyka, MarekBrudnicki, AndrzejMourão, EliseteBruno, Selma SousaMoustakas, LouisBruzzaniti, PlacidoMoya Higueras, JorgeBryndal, AleksandraMoyo, GugulethuBrzeziński, MichałMroskova, SlavkaBućan, KajoMsellati, PhilippeBuchhorn, ReinerMuehlan, HolgerBucur, Sorana MariaMuehlbacher, TobiasBueno De Mesquita, Paul JacobMugarab Samedi, VeronicaBueno, Nassib B.Muller, Jean-baptisteBuhrman, Jason S.Munajat, IsmailBujak, ZbigniewMunir, FouziaBuksnyte–Marmiene, LoretaMuñoz, RickyBulathwatta, AsankaMuñoz-Úbeda, MónicaBulfamante, Antonio MarioMuntean, AlexandrinaBulum, AntonioMuntele Hendreş, DanielaBumin, GoncaMuntianu, Vlad-AlexandruBunc, VaclavMur, SébastienBuonsenso, DaniloMuresan, Gabriela MihaelaBurak, M. FurkanMurillo-Llorente, Maria TeresaBuratta, LiviaMuro, ManuelBurcu Ersöz, AlanMurphy, Andrew J.Burge, Kathryn Y.Murphy, Cara M.Burgos, HéctorMurray, Jane M.Burlui, AlexandraMurray-Stewart,TracyBurugupalli, SatvikaMusso, PasqualeBusoni, VeronicaMustafa, MohammedBustorff-Silva, Joaquim MurrayMustakov, Tihomir B.Bustos, EdsonMuthiah, ArunachalamBuszkiewicz, JamesMuzzi, EnricoButera, AndreaN. Booth, JosieButt, IrfanNa’ara, ShorookButter, SarahNaeem, AsifBüttner-Teleagă, AntjeNagano, ChinaBuzzi, MarinaNagao, Prescilla EmyCaenazzo, LucianaNagoshi, MakotoCagetti, Maria GraziaNagovitsyn, Roman S.Caglic, IztokNahajowski, MarekCainelli, ElisaNaik, SunilCalafiore, DarioNajafi, NadiaCalcagni, GiulioNakaji, KonosukeCalcaterra, ValeriaNakamura, ShotaCaldeira, ElieteNakamura, TadashiCalder, SamuelNakamura, TomokiCaleya, Antonia M.Nakashima, HirotakaCalle, Mariana C.Nakayama, EnriCalvert, MaeganNalli, GiacomoCameron, Kate L.Nalos, MarekCamia, MichelaNam, So-HyunCamoni, NicoleNapoli, FlaviaCampbell, Janine AnneNappi, AnnaritaCamporesi, AnnaNarvey, MichaelCampos, MariaNarzisi, AntonioCampos-Jara, ChristianNascimento, Bruno R.Campus, GuglielmoNasef, NehadCañas, MaríaNaskar, ShovanCanavera, Kristin E.Nathans, LauraCanavese, FedericoNavarro, SergioCancer, AliceNavarro-Alvarez, NaluCanciani, ElenaNavarro-Jiménez, EduardoCanevari, Frank RikkiNavarro-Soria, IgnasiCangussu, Maria C. T.Nava-Zavala, Arnulfo HernanCannarozzo, GiovanniNawrocka, AgnieszkaCannataro, RobertoNayrac, ManonCantero Garlito, Pablo A.Neema, ShekharCantero-Sánchez, Francisco-JavierNegovetić-Vranić, DubravkaCao, ShisongNeil, NicoleCao, WenzhenNemeth, ZsoltCapel, CyrilleNeruntarat, ChairatCapoluongo, NicolinaNesovic Ostojic, JelenaCapote-Puente, RaúlNeubauer, EvaCappella, AnnalisaNewman, StephenCarandang, Rogie RoyceNeymotin, FlorenceCaravaca, Luis AndreuNg, Ting KinCarbajo-Lozoya, JavierNg’ombe, John N.Carbone, Elvira AnnaNgatchou-Wandji, JosephCardero-Durán, M. De Los ÁngelesNi’matuzahroh, Ni’matuzahrohCardoso, MiguelNicholas, MariaCaricato, MarcoNicita, FabianaCarlo, Waldemar A.Nicole, DargueCarmo, CláudiaNielsen, Bettina NygaardCarmona-Barrientos, InesNiermeyer, Susan N.Carneiro, Sueli Coelho Da SilvaNieva, SilviaCarney, Paul R.Niikawa, HiromichiCarolin, SobekNikhil, PillaiCarp, MadalinaNikova, Alexandrina S.Carrilho, EuniceNilsson, AnnaCarrillo-Díaz, MaríaNinchoji, TakeshiCarter, BernieNiseteo, TenaCartujano-Barrera, FranciscoNishi, AkihiroCarvajal Monroy, Paola L.Nishimura, KojiCarvalho, HumbertoNitzan, DoritCarvalho, Pedro B.Nixon, GraemeCasamassimo, Paul S.Nobre, JoanaCasanovas, CarmenNoelia, Navarro GómezCasique, IreneNoel-MacDonnell, Janelle R.Cassioli, EmanueleNoordam, CeesCastaño-Osorio, JhonNordin, PerCastelein, René M.Nori, AnnapurnaCastellani, ChristophNoritz, GareyCastellano, OrlandoNosetti, LuanaCastells, MatíasNota, AlessandroCastro, HenriqueNovák, JanCatalano, HorațiuNoviello, CarmineCataldo, IlariaNovikova, IrinaCatarino, HelenaNovotny, Nathan M.Catela, DavidNovotny, TomasCater, Daniel T.Nowak, RafalCatherine, GireNtolkeras, GeorgiosČatipović, MarijaNugraha, RoniCatsman-Berrevoets, Coriene E.Nunes, CristinaCaturano, AlfredoNúñez Angulo, BeatrizCaurcel Cara, María JesúsNúñez Enríquez, OscarCavaggioni, LucaNurhasanah, FaridaCavalcante Neto, Jorge LopesNuvvula, SivakumarCavaleri, DanieleNuzzolese, EmilioCavaliere, AnnachiaraNyankovskyy, SerhiyCavigioli, FrancescoO’Brien, EileenCaylka, EmrahO’Brien, Nicole FortierCeballos-Laita, LuisO’Halloran, KatrinaCecilia, Raul RuizOberhelman, SaraCejudo, AntonioObrero-Gaitán, EstebanCeliane, Rey-CasserlyObrycki, ŁukaszCemali, MustafaOctavius, GilbertCera, NicolettaOgodescu, EmiliaCeranoglu, T. AtillaOgrodowczyk, AnnaCeraso, AnnaOgunsakin, RopoCeratto, SimoneOh, HoureiCerbara, LoredanaOh, Je HyeokCernadas, JosefinaOh, JinaCernasev, AlinaOhbuchi, ToyoakiCesanelli, LeonardoOhta, KazuhideCetin, BenhurOkafor, FlorenceCeu MacHado, MariaOkazaki, TadaharuChagas, Daniel Das VirgensOkui, TasukuChan, JeffreyOlah, AttilaChandel, AmitOlang, BeheshtehChandra, Manju M.Oldrati, ViolaChandra, SharatOlecká, IvanaChandran, SureshOlicker, Arielle L.Chang, Elizabeth Ya-HuiOliván-Gonzalvo, GonzaloChang, Jui-HsingOlivieri, GiulioChang, Junn-LiangOlomu, IsokenChang, Tu-HsuanOlszanecka-Glinianowicz, MagdalenaChang, Wei-HsiangOng, ShuhwaChang, YaolungOnofrei, VivianaChang, YuanmayOoi, Delicia Shu QinChang, Yuan-YenOpara, Nnennaya U.Chang, Yu-ChanOperto, Francesca FeliciaChang, Yun-HsuanOpydo-Szymaczek, JustynaChapron, ThibautOrovou, EiriniCharitou, SophiaOrtega Toro, EnriqueCharkaluk, Marie-LaureOrtiz-Marrón, HonoratoChaturvedi, KaushalendraOrtiz-Tallo, MargaritaChatzidimitriou, DimitriosOrtiz-Toquero, SaraChaudhary, ShipraOrton, JaneChauhan, DhavalOsborne, RichardChawla, JasneekOstermeier, EmmaChelkeba, LegeseOstrowska, AureliaChen, Chi-NienOstrowska, BożenaChen, Chung-MingOswald, Kaitlin A.Chen, Hsiu-LinOtani, KojiChen, I. HuaOtani, NorioChen, Jia YuhOttarsdottir, UnnurChen, JunxiangOtth, MariaChen, Kuo-TaiOttóffy, GáborChen, Li-WenOue, TakaharuChen, MingOuwehand, KimChen, RebeccaOuyang, Yan-QiongChen, Ru-SiOzbič, MartinaChen, SisiÖzdemir, SametChen, Si-TongÖzen, GökmenChen, Wei-TiÖzkan, EsmaCheney, Ann M.Paal, PeterCheng, Chi-HuiPaavilainen, EijaCheng, Chun-AnPace, AnnalisaCheng, JiaojiaoPadial-Ruz, RosarioCheng, WeiPaduraru, LuminitaCherepov, EvgeniyPaez-Moguer, JoaquinChern, AlexanderPagano, StefanoChethan, K. N.Pagé, GwenaëlCheung, Johnson Chun-SingPagliarino, ElenaChevidikunnan, Mohamed FaisalPahlman, MagnusChiara, Maria DiPahor, DusicaChigbu, De Gaulle I.Pal, AjayChim, Li ManPałasz, ArturChimenz, RobertoPaleg, GinnyChimoriya, RiteshPalinkas, MarceloChing, Chi KeongPalli, EleniChing-Chung, TsaiPalma, SilviaChiou, Chou-PingPalmer, April L.Chiracu, AlinaPalmisciano, PaoloChirico, IlariaPalomo-Carrión, RocioChiti, Maria-CostanzaPalomo-Osuna, JeniferChitty, Kate M.Palucci-Vieira, LuizChmielewski, PrzemyslawPalumbi, RobertoChmielik, Lechosław PawelPanagopoulos, DimitriosCho, Jeong-ilPanganiban, JenniferCho, KihoPanicucci, ChiaraCho, Mi-KyoungPapadimitriou, AnastasiosCho, VanessaPapadopoulos, DimitriosChoate, PeterPapageorgiou, Ismini E.Choi, Hee-YoungPapamitsou, TheodoraChoi, Jong-WoonPapotto, GiacomoChoi, Joon HyukParadowska-Stolarz, AnnaChoi, Mi-YoungParastatidou, StavroulaChoi, NayaParat, SumeshChoi, Tae-SooPareek, GautamChoonara, ImtiPareja, Jennifer MunozChou, Yen YinPark, Eun-YoungChovancová, ZitaPark, JusikChrastina, JanPark, Shin JunChristofaro, Diego Giulliano DestroPark, YoungheeChrobok, ViktorParola, AnnaChrysanthakopoulos, Nikolaos A.Parra, Antonio FernándezChu, Nain-FengParra, DenisseChungsomprasong, PaweenaParra-Pérez, Lizeth GuadalupeCicchella, AntonioParrish, RichardCiećwież, SylwesterParschat, KatjaCieślik, BłażejParsons, Lauren N.Cimbanassi, StefaniaPascual, Juan C.Cinicola, Bianca LauraPasqualetti, PatrizioCiodaro, FrancescoPassanisi, StefanoCioffi, AndreaPassmore, ElyseCirio, SilviaPastor-Pons, InakiCiubara, AnamariaPatel, BrijeshkumarCiuca, CristinaPatel, ChhayaČižman, MilanPatel, JilenClapton, GaryPatel, MaulinClark, M. DianePatel, NehaCoca, Juan R.Pathak, DhrubaCockerton, TraceyPathak, RajivCoelho, AnaPatón, Rubén NavarroCofaru, Nicolae-FlorinPatricia Correa, De FariaCoffey, NiamhPauls, FranzCohen, Arieh SierraPăun, DanCohen, Nathaniel AvivPavelescu, CarmenCohen, Philip N.Pavelka, KarelColella-Santos, Maria FranciscaPavone, MartinoČolović, ZavišaPavone, VitoCondello, IgnazioPavoni, MatteoCondessa, Isabel CabritaPawlaczyk-Kamienska, TamaraConsales, AlessandraPaz Lourido, BertaConstain Moreno, Gustavo EduardoPearce, Thomas M.Constantinescu, AlexandruPease, Anna S.Constantini, ShlomiPecci-Lloret, Miguel R.Constantinou, MariosPecor, Keith W.Convento, MarciaPeden, MargieConvissar, Robert A.Peeples, EricCooley, FrancesPegado, ElsaCools, WouterPeitsidis, PanagiotisCooney, RachelPek, Jen HengCopersino, MarcPekçetin, SerkanCoppens, PatrickPeláez-Ballestas, IngrisCoppola, GianlucaPelegrín, AntoniaCorcuff, Jean BenoîtPellegrini, Massimiliano MatteoCordeiro, RitaPellicano, RinaldoCordellat Marzal, AnaPeluso, FrancescaCorral Liria, InmaculadaPeñate, WenceslaoCorreia, TâniaPenna, Maria PietronillaCorrell, LynniePereira, ArmandaCortelazzo, SergioPereira, Silvana AlvesCortese, AntonioPereira, SujithCortes-Ramirez, JavierPereira-da-Silva, LuísCosta, Anna MariaPerenc, LidiaCosta, LorenzoPeres, Nalu T. A.Costa, Luciane R.Pérez De Sevilla, Guillermo GarcíaCosta, Roberto FernandesPérez-Ardanaz, BibianaCostabile, AngelaPerez-Burriel, MarcCostantino, AndreaPérez-Cruz, MiriamCostanzo, SaraPérez-Jover, VirtudesCotugno, NicolaPérez-Marín, MariánCouch, James R.Perez-Solans, BelenCouchonnal, EduardoPérez-Torres, IsraelCourvoisier, AurélienPergialiotis, VasiliosCoussens, ScottPerisano, CarloCovantev, SergheiPerri, DamianoCox, Des W.Perry, AudreyCoyle, Patricia K.Perry, John D.Cozzi, DenisPersichetti, AlessandraCradock, GeraldPertea, MihaelaCRAIU, MihaiPesaturo, KimberlyCrippa, MatteoPetat, HortenseCrisol Moya, EmilioPetca, AidaCron, Randy Q.Peters, KathCruz, JoanaPetersen, ClausCruz-Ramos, MarlidPethő, ÁkosCruz-Santos, AnabelaPetrariu, Florin-DumitruCsima, MelindaPetrauskiene, SandraCuartero-Castañer, Maria ElenaPetridis, LeonidasCuberos, Ramón ChacónPetrigna, LucaCucchetti, AlessandroPetrocheilou, ArgyriCucinotta, FrancescaPetrova, GerganaCui, XinPetrovičová, IdaCunha-Oliveira, AlietePetsios, KonstantinosCunitz, KatrinPeyer, KarissaCunningham, MelodyPfister, Katie M.Cunningham, Natoshia R.Phad, Nilkant S.Curle, DeirdrePhantumvanit, PrathipCurto, AdrianPiatkowska, MonikaCushley, Laura N.Piau, CarolineCybulska, AnnaPienaar, Anita ElizabethCynarski, Wojciech J.Pietrasanta, CarloCypriano, MonicaPiirainen, PanuCzerwińska, JoannaPilar-Orive, Francisco JavierD’Agostino, SilviaPillai, NishithaD’Onofrio, GraziaPilmane, MāraD’Souza, RussellPineda-Espejel, Heriberto AntonioDa Cruz Fernandes, LucienePinero-Pinto, ElenaDa Silva Batista, Marco AlexandrePinto, AlexDa Silva, Danilo Grünig HumbertoPinto-Simões, BelindaDa Silva, José AparecidoPiozzi, Guglielmo NiccolòDa Silva, KeithPiri, RezaDagklis, Themistoklis I.Pirrolas, Olga Alexandra ChinitaDagla, MariaPisano, FrancescaDahdah, NagibPisano, SimoneDahlof, GoranPiscitelli, DanieleDal Pupo, JulianoPiskunov, AlexeyDamiano, Diane L.Pitti, Julio AlvarezDampier, CarltonPitton Rissardo, JamirDang, ShaonongPitucha, GrzegorzDanielescu, CiprianPiva, GiovanniDanielewicz, AnnaPizzuto, Maresca AttardDarby, KevinPlagens-Rotman, KatarzynaDargue, NicolePlantaz, DominiqueDariel, AnnePlaza, GuillermoDas Neves, Lucimara TeixeiraPlaza-Díaz, JulioDasgupta, SohamPlourde, BruceDaskalaki, EvangeliaPlut, DomenDaubmann, AnnePneumaticos, Spiros G.Davies, AdamPochstein, FlorianDavis, KathleenPodder, ShreyaDay, AndrewPoddighe, DimitriDe Barros Silva, Paulo GoberlânioPodgórska-Bednarz, JustynaDe Berardis, DomenicoPoggi, GeraldinaDe Bernardi, FrancescaPogorelić, ZenonDe Giambattista, ConcettaPojskic, MirzaDe Kruijff, InekePokorska-Niewiada, KamilaDe La Cruz-Mosso, UlisesPokorska-Śpiewak, Mariade la Cruz-Torres, BlancaPokrovskaia, Nadezhda N.De Lazzari, ClaudioPolechoński, JacekDe Re, ValliPolgar, NenadDe Rosa, VincenzoPolic, BrankaDe Roubaix, AmyPollastro, CarlaDe Sanctis, Juan BautistaPombo, AndréDe Silva, P. Mangala C.S.Pomeroy, Jeremy J.De Ville De Goyet, MaëllePons Escoda, AlbertDe Vries, Linda SimonePop, OvidiuDębska, MarzenaPopa, AlexandruDedeken, LaurencePopa, Alina DeliaDefresne, PierrePope, FrancisDeguise, Marc-OlivierPoppers, David M.Dekanic, AndreaPorczyńska-Ciszewska, AnnaDel Barrio Gándara, M VictoriaPortelli, MarcoDel Campo, MiguelPortinaro, NicolaDel Pozo De La Calle, SusanaPorwal, AmitDella Corte, MarcelloPosa, AlessandroDeLuca, Stephanie C.Posar, AnnioDemareva, ValeriiaPosner, JonathanDembowska-Baginska, BozennaPoston, BrachDemey, BaptistePotměšilová, PetraDemir, SelmaPoulia, Kalliopi AnnaDemirci, EsraPourshahidi, KirstyDemirdag, Tugba BedirPower, CarmenDenaro, LucaPower, RosalieDeng, WeiyangPoznanski, PiotrDenis, FredericPozo-Rico, TeresaDepetris, NadiaPrakasam, RamachandranDer Ham, Ineke J.M. VanPrasadh, SomasundaramDerwich, KatarzynaPrasertsukdee, SaipinDerwich, MarcinPreda, CamillaDesai, SaralPreda, Silviu DanielDeshwal, HimanshuPreston, LynnDeUgarte, Daniel A.Printza, Nikoleta G.Dev, RishabhPritchard, K. A.Devaney, CarmelPriyam, JaniDevine, JaimeProkopiak, AnnaDevlin, HughPruccoli, JacopoDharmaraj, RajmohanPrzestrzelska, MonikaDhawan, GauravPsychogios, KlearchosDhungana Sainju, KarlaPulling Kuhn, AnnDi Giorgio, GianniPunjani, NeelamDiao, Pei-HuangPurpura, GiuliaDiarbakerli, EliasPušnik, IgorDíaz-Álvarez, AgustínPutri, YubbuDíaz-Castro, LinaPyke-Grimm, Kimberly A.Dibello, DanielaQanash, HusamDickinson, SarahQin, Kirby R.Diehl, KatharinaQuaresima, PaolaDíez López, IgnacioQuilling, EikeDíez, EmilianoQuinodoz, MathieuDİKEÇ, GülQuitadamo, Pasqua AnnaDilts, JenniferQureshi, Ambrina A.Dincher, AndreaQureshi, Mosarrat J.Ding, YiQvist, NielsDiogo, EliseteRabinowitz, Loren GallerDipalma, GiannaRachmawati, DessyDiplomatico, MarioRachwani, JayaDirksen, DieterRaczkiewicz, DorotaDisouky, AhmedRaczkowska, EwaDistefano, RebeccaRadmilović-Radjenović, MarijaDivaris, KimonRaducan, ViorelDjakow, JanaRadzki, DominikDjuricic, IvanaRaffaele, AlessandroDmytriiev, DmytroRafiei Vardanjani, AhmadDo Prado, RogérioRaghavan, SreevatsanDobrzynska, MalgorzataRaghu, Vikram K.Dolic, KresimirRaghuram, KaminiDolivo, DavidRahbek, OleDombrecht, LaureRahelić, ValentinaDomingues Ferreira da Cruz, Mário RuiRahotă, Răzvan GeorgeDomínguez Herrera, RaulRaigani, SiavashDomínguez-Lloria, SaraRaiola, GaetanoDomini, MarcelloRajendra Rao, SumithaDönmez, Muhammet İrfanRajsic, SasaDonner, DavideRam, DianaDores, Artemisa RochaRamalho, Teodorico C.Dou, DiyaRaman, SnehaDoucette, John T.Rameckers, Eugène A.A.Dovnik, AndrazRamirez‐Amador, VeliaDowell, Richard C.Ramklint, MiaDragonieri, SilvanoRamos, AmandaDresing, KlausRamos, AnaDriessen, GeertRamos-Vidal, IgnacioDruica, ElenaRando-Cueto, DoloresDrummond-Lewis, Sasha R.Ranganathan, SarangarajanDryga, AnatolyRangel-Ledezma, Yunuen SocorroDu, MeijunRangon, Claire-MarieDuan, ZongshuanRanka Stirn, KranjcDube, AdieleRao, Suchetha S.Duchnowski, PiotrRaphaelli Nahás-Scocate, Ana CarlaDuci, MiriamRapisarda, FilippoDueñas, Jorge-ManuelRapp, Paul E.Dueppers, PhilipRasulić, LukasDugar, SiddharthRay, Anish K.Duică, LaviniaRecker, FlorianDumitraşcu, TraianRedaelli, SimoneDumitrescu, Alina V.Redondo-Gutiérrez, LauraDumitru, Irina MagdalenaRega, AngeloDuncan, Emma L.Rehman, LaureneDunican, Eleanor M.Reimer, AntoniaDunker, Karin Louise LenzReinoso-Cobo, AndresDurán-Bouza, MontserratReisinho, Maria Da ConceiçãoDurdik, PeterReiss, Allison B.Duse, CarmenRelva, Inês CarvalhoDusser, PerrineRenner, JaneDutton, Gordon NealeResch, BernhardDuursma, Elisabeth E.Resende, Briseida DogoDwivedi, MiteshRevel-Vilk, ShoshanaDworkin, Paul H.Rey Galan, CorsinoDyer, Laura A.Reynolds, Barry LeeDylag, Andrew M.Rhee, IsaacE. Krause, JaneRhee, Seung-YoonEigentler, AndreasRhine, William D.Ek, StaffanRhodes, Diane McDanielEl Meligy, OmarRibaudo, JulieElhamouly, YasmineRibeiro, IsildaElia, MaurizioRiccio, Maria PiaEliasz, WojciechRichard, CelineElliman, DavidRico-Díaz, JavierElsherbiny, NehalRidao, PilarEltelety, Ahmad MohamedRidley, SusanEmre, IlhanRikos, NikolaosEnciu, OctavianRimbert, AntoineEndo, KaoriRinaldi, PasqualeEndoh, DaijiRinta, TiijaEngdahl, IngridRisica, Patricia MarkhamEngel, CorinnaRitter, AlexanderEngelhardt, Thomas E.Riva, GiuseppeEnninghorst, NatalieRivera-Picón, CristinaEntleitner-Phleps, ChristineRivero, MagdaEpstein, ShulamitRoberts, Anne M.Erber, RalfRoberts, Michael W.Eren, BülentRobertson, LouiseErgun, OrkanRobinson, RisaErickson-Owens, DebraRobitaille, EricErmund, AnnaRoccella, MicheleErnesto, Cortés-CastellRocchi, AlessiaErtl, TiborRocha, Sabrina G. M. O.Ertl-Wagner, Birgit B.Rodrigues, Carina De FátimaEscobio-Prieto, IsabelRodrigues, DanielaEscolano Pérez, ElenaRodrigues, Daniela O. W.Escortell Sánchez, RaquelRodrigues, JorgeEspinosa, SusanaRodrigues, Vandilson PinheiroEspinosa-Sanchez, Juan ManuelRodrigues, VitorEsposito, GianlucaRodríguez Testal, Juan FranciscoEsposito, PasqualeRodriguez, ChristinaEspoz-Lazo, SebastiánRodríguez, Isaías GarcíaEsteban, LauraRodríguez-Arce, JorgeEswar, KandaswamyRodríguez-Archilla, AlbertoEvangelista, Danielle R.Rodríguez-Hernández, Antonio F.Evans, Amanda M.Rodríguez-Hurtado, DianaEvanson, Nathan K.Rodríguez-Noriega, EduardoEwais, TatjanaRodríguez-Pérez, José ManuelEyskens, FrancoisRoehr, Charles C.Fabbris, CristoforoRoganovic, JelenaFachada, Miguel ÂngeloRoginsky, EfratFailo, AlessandroRogowska, AleksandraFainardi, ValentinaRoh, Tae HoonFaísca, LuísRöhrlich, Pierre SimonFalco, JacopoRój, JustynaFalcón Miguel, DavidRojano, ElenaFan, QiaoRojas, Antonio Daniel GarcíaFang, WangRojas, Wilfredo De JesúsFantone, SoniaRojas-Russell, M. E.Farì, GiacomoRoka, AttilaFaria, VandaRolandsdotter, HelenaFariña, Ana ClaraRolla, GiovanniFarokhi, Moshtagh R.Rollè, LucaFarr, SebastianRomandini, AlessandraFascetti Leon, FrancescoRomaniuk, PiotrFaulkner, Melissa SpeziaRomano, CorradoFaustino-Silva, Daniel DemétrioRomano, MarcelloFavara-Scacco, CinziaRomero Martínez, Sonia J.Favretto, DonataRomero-Velarde, EnriqueFederico, FrancescaRonnenberg, MeganFeduniw, StepanRosas-Vargas, HaydeéFees, Bronwyn S.Roscoe, Clare M. P.Feier, Andrei MarianRosenbaum, DieterFekonja, AnitaRosenbohm, AngelaFeldman, KeithRosenstock, TizianFelix, MarkRospleszcz, SusanneFeloukidis, ChristosRosser Limiñana, AnaFeng, JunRossetti, AndrewFennell, CurtisRossi, AdrianaFenton, Tanis R.Röszer, TamásFeraco, PaolaRowe, Meredith L.Ferkol, Thomas W.Różańska, SylwiaFernandes, Patrícia Raquel SilvaRóżańska-Walędziak, Anna MariaFernández Medina, Isabel MaríaRozensztrauch, AnnaFernández-Alvarez, ClaraRozmiarek, MateuszFernández-Barrera, Miguel ÁngelRubín, José ManuelFernández-Prados, Juan SebastiánRudrappa, MohanFernández-Sáez, JoseRugytė, DanguolėFernando Mourão, CarlosRugyte, Danguole CeslavaFerraro, AntoninoRuin, SebastianFerraro, Thomas N.Ruiz-Rodríguez, IvánFerreira, PaulaRuiz-Ruano García, Ana MaríaFerreira, PauloRungratsameetaweemana, NuttidaFerreira-Santos, FernandoRupp, MarkusFerrerós Pagès, CarlaRupprecht, MartinFerretti, GeraldRusciano, DarioFesslova, VlastaRusmini, MartaFialkowski, Elizabeth A.Russell, MarionFicial, BenjamimRussu, Octav MariusField, MichaelRustico, LorenzoField, Stephanie C.Rusu, Alina SimonaFigura, MonikaRusu, LigiaFilip, FlorinRusy, LynnFilipiak, SaraRutherford, MarionFilipović-Grčić, BorisRutkauskaite, RenataFilocamo, GiovanniRyabov, IgorFiner, NeilRyan, KathleenFinken, Martijn J. J.Ryskulova, AselFinlayson, JanetSaba, FakhredinFinley, G. AllenSabico, ShaunFinotti, MicheleSabiniewicz, RobertFinsterer, JosefSabri, Mohamad FazliFionda, BrunoSacchi, ChiaraFiore, MicheleSachmechi, IssacFiori, SimonaSaez De Ocariz-Gutierrez, Marimar M.Fisberg, MauroSaez, IkerFischer, Gilberto B.Safder, OsamaFischer, Martin H.Saft, ScottFischer, TatjanaSagar, PremFish, JenniferSagiv, EyalFisher, HelenSagone, ElisabettaFitzpatrick, TiffanyŞahin, ErtuğrulFlack, John M.Saito, JumpeiFlapper, Walter J.Sakamoto, TatsunoriFlegner, PatrikSakellari, EvanthiaFlores-Pliego, ArturoSalah, Nouran YousefFloríndez, Lucía I.Salama, Fouad S.Florio, EleonoraSalami, FiroozFogacci, FedericaSalamon, Katherine S.Fogel, YaelSalavera Bordás, CarlosFominiene, Vilija BiteSaldaña-García, NataliaFoote, Janet A.Saleh, NagwanFord, SuzanneSalerno, ClaudiaForero-Castro, MaribelSalido-Vallejo, RafaelFornara, RobertoSalinas-Roca, BlancaForrest, ClaireSalomão, José Francisco M.Forsberg, Birger CarlSalome, SerenaFoster, JenniferSalvatore, Christine M.Foti, GiovanniSalvio, GianmariaFotoulaki, MariaSamadi, Sayyed AliFrade, João Manuel GraçaSamango‐Sprouse, Carole A.Fradkin, YuliSamborski, WlodzimierzFraijo Sing, SilviaSampaio, FranciscoFrancavilla, MariantoniettaSamples, Stefani M.Franceschini, FabrizioSamudra, PreetiFrancia, GuadalupeSánchez González, Juan LuisFrancis Borgio, J.Sánchez González, María-CarmenFranck, LindaSánchez Loyo, Luis MiguelFranco, SusanaSánchez Ojeda, María AngustiasFranke, DorisSanchez, IreneFranzese, AdrianaSanchez, LeonorFraser, LornaSánchez-Alberola, FranciscoFrassanito, AntonellaSanchez-Diaz, ManuelFraunberger, ErikSánchez-López, PilarFreitas-Almeida, Angela CorreaSánchez-Romero, Elisa I.Friedlander, LisaSandoval-Obando, EduardoFriedman, Rick A.Sangiorgio, Sophia N.Frosch, MichaelSani, Ana IsabelFrosolini, AndreaSankaran, DeepikaFu, Chun-MinSano, YujiroFu, Ren-HueiSant’Anna, ClemaxFu, TingSantacaterina, FabioFuchs, JuriSantamaría-Peláez, MirianFuente-Hernández, J.Santi, KristiFujak, AlbertSantiago-Rodríguez, EfraínFujii, YoshimitsuSantilli, ManlioFujimoto, AyatakaSantos, Ana IsabelFukuda, MotokiSantos, RuteFulceri, FrancescaSantos, SofiaFunuyet-Salas, JesúsSantric Milicevic, MilenaGabr, SamiSapienza, MarcoGabrieli, GiulioSarafidis, KosmasGabryelska, AgataSaraiva, LindaGadepalli, ChaitanyaSarantopoulos, George PeterGafencu, MihaiSarapultsev, AlexeyGage, Nicholas A.Saravi, BabakGailite, JurgitaSarbu, IoanGajdzica, ZenonSardana, DiveshGalán, MaríaSarhan, AdnanGalan-Lopez, PabloSarier, MehmetGalatti, LarissaSarmet, MaxGaliatsatos, AristidisSartor-Harada, AndresaGaliniak, SabinaSartucci, FerdinandoGallardo Montes, Carmen Del PilarSarul, MichalGallardo, Nuria E.Sarul, MichałGallazzi, EnricoSasaki, HideyukiGallegos-Cázares, LeticiaSassu, RalucaGallone, GiovanniSathe, MeghanaGálvez Espinoza, PatriciaSattler, Kierra M. P.Gambazza, SimoneSava-Rosianu, RuxandraGameiro, FáimaSavin, CarmenGamze Gokalp, GamzeSavory, NicolaGandhi, RaghuSawaya, YoheiGanguly, AbhrajitScala, IrisGao, JieScariot, Pedro Paulo MenezesGao, PengSchäfer, Frank-MattiasGao, XuesongSchäffeler, NorbertGaragiola, UmbertoSchalamon, JohannesGarayzábal-Heinze, ElenaScheinemann, KatrinGarcia De Avila, Marla AndréiaScher, MarkGarcía Iglesias, DanielScheuer, ClaudeGarcia Laborda, JesusSchmedding, AndreaGarcía, Andrea GutiérrezSchmidt, PeterGarcía-Galbis, Manuel ReigSchmittenbecher, Peter P.García-García, SergioSchneider, Camille R.Garcia-Gasca, AlejandraScholbach, ThomasGarcía-Medrano, BelénScholz, Konstantin JohannesGarcía-Mendoza, María Del CarmenSchuler Faccini, LaviniaGarcía-Morales, ClaudiaSchuler, RahelGarcía-Moro, Francisco JoséSchulte, AndreasGarcía-Muñoz Rodrigo, FerminSchütz, KatharinaGarcia-Munoz, CristinaSchwaberger, BernhardGarcía-Pola, María JoséSchwaiger, MichaelGardner, Susan T.Schweizer, CelineGarner, AngieScibelli, FrancescoGarrido Martinez de Salazar, FelipeScolieri, PalmaGarvie, Patricia A.Scott, DebbieGash, HughScottoline, BrianGasparella, PaoloSeaby, Eleanor G.Gastaldi, FrancescaSedláček, PetrGavic, LidiaSees, Julieanne P.Gawlinski, PawelSegar, Jeffrey L.Gawryjołek, JuliaSegù, MarziaGazerani, ParisaSelva, David M.Gazit, RoiSerban, DragosGeetha, ThangiahSergentanis, Theodoros N.Gelabert, Sebastià VergerSerna, CristinaGelemanović, AndreaSerra, MariaGellel, Adrian-MarioSetanen, SirkkuGennaro, Giovanni Luigi DiSetiawan, Arlette SuzyGenovese, DarioSeweryn, ArturGenovese, ElisabettaSferrazza Papa, Giuseppe FrancescoGentil, FernandaShaffer, MarkGentileschi, PaoloShafiq, SaimaGentil-Gutiérrez, AnaShah, Manish N.Gentle-Genitty, CarolynShah, PayalGenyk, Yuri S.Shahali, ShadabGeorge, KoumantakisShaheen, BassamGeorge-Puskar, AnnieShaman, NicholasGeppe, NataliaShamionov, RailGerlach, JenniferShan, LingpengGershoff, ElizabethShang, GuowenGerson, TrevorShanthikumar, ShivanthanGhanamah, RafatSharma, AnirudhGhele, PetraSharma, AnudeepaGheorghe, Dorin-NicolaeShe, XinshuGheorghita, DanielaShek, DmitriiGhezzi, MicheleShenoy, SunainaGhezzo, AlessandroSherriff, JennyGhorbani, SaeedShi, Hon-YiGhosh, ArunavaShiao, An-SueyGiacomelli, MauroShierk, AngelaGiacomo, Andrea DeShim, Joon W.Giannattasio, SilviaShin, KyuleeGibler, Robert C.Shinya, AkikazuGieysztor, EwaShioji, NaohiroGilbertson, LauraShirota, YuichiroGill, KamilShkodra, MorenaGillentine, MadelynShohel, M. Mahruf C.Ginani, Verônica CortezShoji, HiromichiGiraudo, FrancoShort, LeonieGiraudo, HélèneShumakova, AntoninaGisbert, Javier P.Sibindi, AtheniaGitirana, MarceloSidiropoulos, KonstantinosGiuca, Maria RitaSiebers, RobGiuliano, Antonio Francesco MariaSiegel, Robert M.Głąbska, DominikaSierra, Javier OrtuñoGladstone, MelissaSierra-Campos, ErickGlavaš, DuškaSignorelli, ChristinaGlažar, IrenaSik Goh, TaeGlowacki, JakubSikalidis, AngelosGlumac, SandroSila, SaraGmaj, BartłomiejŠileikienė, RimaGodambe, SunitSilén-Lipponen, MarjaGodínez-Chaparro, BeatrizSilișteanu, SinzianaGodoy-Cumillaf, AndrésSilka, LindaGoetschalckx, MiekeSilva, Elisabete P.Gois, Glayciane CostaSilva, IvaldoGoldschmidt, ImekeSilva, Luiz FelipeGoldstein, Allan M.Silva, Maria SebastianaGolkocheva-Markova, ElitsaSilva, MarianaGómez Velázquez, Fabiola R.Simeon, James C.Gómez, Nidia N.Simione, MegGomez-Baya, DiegoSimkovich, Suzanne M.Gonçalves Neves, BeatrizSimoes, Jose A. O.Goncalves, AurélieSimoës-Perlant, AurélieGoncalves, Widjane Sheila FerreiraSimon, Anne-LaureGonella, SilviaȘimon, MărioaraGonzalez Ruiz, Jose MariaSimon, ThorstenGonzález, PascualSimonini, AlessandroGonzález-Aragón Pineda, Álvaro EdgarSimos, GregorisGonzález-Ródenas, JoaquínSimpson, EdwardGonzález-Valero, GabrielSimunovic, MarkoGoodwin, MichaelaSingh, AmbrishGooty, Vasu D.Singh, AmitGormley, Andrew K.Singh, BrajeshGosak, LucijaSingh, GoutamGossrau, GudrunSingh, GurdeepGottliebsen, MartinSingh, JitendraGozalo Delgado, MargaritaSingh, KailashGraça, Flávia AparecidaSinghi, Anil KumarGradova, MargaretSinghvi, AditiGraeber, SimonSiniscalchi, AntonioGraham, TiffanySiniscalco, DarioGrajek, MateuszSinning, ChristophGrammatico, PaolaSinopoli, AlessandraGrammatikopoulou, Maria G.Sirico, DomenicoGranberg, DanSisi, TaoGrasa, Carlos D.Sisk, BryanGraw, Jan AdriaanSiwicka-Gieroba, DorotaGray, KellySkalski, SebastianGreene‐Woods, AshleySkarżyński, Piotr HenrykGreenhalgh, DavidSkolmowska, DominikaGreenough, AnneSkoulakis, Charalampos E.Grenda, RyszardSkrzypczak, PiotrGriese, MatthiasSlominsky, Pyotr A.Grifoni, GinoSmane, LieneGrigoletto, AlessiaSmereka, JacekGrigoraș, AdrianaSmiley, Natasha PillayGrigorescu, MelissaSmirni, DanielaGrigoriou, Maria E.Smith, Albert F.Grigoriu, Anca-IrinaSmith, AllisonGrilo, MargaridaSmith, CatherineGriniatsos, JohnSmith, Sharon R.Grivas, Theodoros B.Smith-Hicks, ConstanceGros, Catherine-IsabelleSmyczyńska, JoannaGruber–Bzura, Beata M.Snir, SharonGrudzińska, EwaSnowden, JessicaGrujić-Milanović, JelicaSoares‐Oliveira, MiguelGruszfeld, DariuszSobierajski, TomaszGrzela, KatarzynaSocea, BogdanGrzywacz, RenataSocolov, RazvanGualdi-Russo, EmanuelaSodhi, MikuGuardamagna, OrnellaSofologi, MariaGuarina, AngelaSogos, CarlaGuarnieri, RosannaSöğüt, MustafaGuazzaroni, GiulianaSoleman, JehudaGuel-Klein, SafakSolgi, GhasemGuénoun, TamaraSolimini, RenataGuerrero, Ángel L.Solís Sánchez, GonzaloGuerrero-Gironés, JuliaSoltanifar, MohsenGuiducci, LetiziaSong, In GyuGuimaraes, Douglas MagnoSong, KyungchulGuimaraes, HerciliaSormunen, JormaGüner, SenemSosnowska-Sienkiewicz, PatrycjaGur, MichalSoto-Cámara, RaúlGurleyik, DuyguSoto-Sanz, VictoriaGutiérrez, LauraSoto-Varela, RobertoGuzmán-Flores, Juan ManuelSoundappan, Soundappan S. V.Guzman-Quevedo, OmarSousa, Mariana Lopes DeH. Molnár, AndorSpagnuolo, Maria ImmacolataHaak, ThomasSparkle, TanayaHaarbauer-Krupa, JulietSpekker, EleonóraHaas, DorotheaSpennato, PietroHaas, NikolausSpeybroeck, Alexander VanHabek, DubravkoSpiewak, MateuszHabibi, AkhmadSpinelli, MarcoHadi, AmirSpitz, LewisHadžić, VedranSporea, CorinaHaecker, Frank MartinSpreat, ScottHaffez, HeshamSprunt, BethHagelsteen, KristineSpurr, ShelleyHahn, JuergenSrivastava, Kumar ChandanHaid, BernhardSt. Cyr, John A.Hajeer, MohammadStadnicki, AntoniHakami, ZakiStampoltzis, AglaiaHalasi, SzabolcsStankova, MargaritaHalling, CecilieStarobrat, GrzegorzHalvorsen, Cecilia PegelowStarshinova, AnnaHama, SarhangSteenblock, CharlotteHammerl, Jens AndréȘtefan, Paul-AndreiHammoud, Nurah M.Stefaniak, NicolasHamwey, MeghanStefanowicz, JoannaHanak, BrianStein Duker, LeahHanaoka, NozomuStenström, PernillaHand, IvanStephanie, BrosiusHandelman, KennethStephens, CamillaHansmann, SandraStetter, Maria EarmanHanstock, TanyaStock, AnnikaHaq, IramStoian, DanaHaque, ArifulStojek, MagdalenaHardy, KeiranStokke, Jamie L.Harel, DovratStokmans, MiaHariyanto, Timotius IvanStolz, Andrew AbbaHarsanyi, StefanStreuli, Jürg C.Hartanto, AndreeStroebele-Benschop, NanetteHartman, EstherStroth, SannaHarzer, KlausStrub, MarionHasan, AbdulkarimStruck, ManuelHashim, ZiaŠtrumfa, IlzeHashimoto, KazuhikoStrzelecka, JolantaHashimoto, TakashiStubbing, JessicaHassett, AlexanderStupar, DusanHassib, Nehal F.Su, ErikHaveman, Jan WillemSu, MaxwellHayashida, KenjiSuárez-Varela, María M. MoralesHayiou-Thomas, Marianna EmmaSuazo, JoséHayward, R. DavidŠubelj, MajaHaywood, DarrenSubramanian, AnitaHe, ChaoSuchman, EricaHeadrick, JonathonSuchý, JiříHeckmann, MatthiasSuder, AgnieszkaHector, Ralph D.Sukumar, SmithaHegedűs, KatalinŠukys, SauliusHeilmann, FlorianSullivan, Kevin M.Heisinger, StephanSumalla-Cano, SandraHell, Anna KathrinSumitomo, NaofumiHeller, JanoschSumiyoshi, MasatakaHelton, Jesse J.Sun, Kuo-TingHennessey, AlexandraSundov, ZeljkoHensel, Charles H.Suppan, LaurentHerberg, UlrikeŠušnjar, TomislavHerbert, AnthonySuyanto, SuyantoHerbert, JarosławSuzuki, Yuichiro J.Herkenhoff, Marcos EdgarSwain, AnneHermand, EricSwana, Hubert S.Hermann, Kay GeertŚwierczyńska, JustynaHernaiz-Sanchez, AriadnaSypianska, JolantaHernández Cruz, GermánSysoeva, OlgaHernández, AdrianaSzabo, Dan-AlexandruHernández-Padilla, EduardoSzabo, TamasHerrero, ChristinaSzarpak, LukaszHertzmark, EllenSzejko, NataliaHeschl, StefanSzepietowska, KatarzynaHicham, ElmsellemSzulc-Musioł, BeataHicks, ElizabethSzwedowski, DawidHigueras, Jorge MoyaTaber, ChristopherHill, Douglas L.Tachibana, MasayaHiram, RoddyTacke, MoritzHirvonen, JussiTadin, AntonijaHoehl, SebastianTafiadis, DionysiosHofbauer, Thomas M.Tagawa, TsuyoshiHofer, JohannesTagliati, CorradoHogue, GrantTakahashi, MitsuhikoHolland-Cunz, StefanTakahashi, YasuhiroHollis, BruceTakimoto, HidemiHolmström Rising, MalinTalavera, MartaHolzinger, DanielTalero-Gutiérrez, ClaudiaHom, ChristyTalluri, JacopoHomman-Ludiye, JihaneTalovacci, Marie-PierreHonda, ChikaTamada, IkkeiHong, Andrew L.Tamm, Anna-liisaHong, Bo-YoungTan, JonathanHopwood, NickTan, MawHorger, MelissaTanaka, KayoHorhat, Delia IoanaTanaka, NaoroHorman, SandrineTanase, MihaelaHorsburgh, K. AnnTandon, SnehaHorslen, SimonTang, NelsonHosoya, MakotoTanriverdi, Halil IbrahimHoteit, MahaTarallo, LuigiHou, Wai KaiTarazona-Meza, CarlaHouck, PhilipTarca, ElenaHowerton-Fox, AmandaTarcea, MonicaHresko, Michael T.Tashima, ToshihikoHsiang Ning, LukTashiro, AiHsieh, Kuan-YingTasic, DanijelaHsieh, Liang PoTatnell, RuthHsu, Chin-MuTatsi, Elizabeth-BarbaraHsu, Po-ChengTaylor, AlyxHsu, Wen-Wei RobertTaylor, Brittany K.Hu, Wen-pinTaylor, ChristineHuang, CharlesTayyem, ReemaHuang, Chien-ChiaTejedor, Félix MarcosHuang, ChienhsiuTékus, EvaHuang, Hsin-ChunTembhre, Manoj KumarHuang, Hung-YuTemiz, Selami AykutHuang, JueTemsah, Mohamad-HaniHuang, LihongTeng, Ru-JengHuang, Shi-HaoTennant, MarcHuang, Tse-YangTeressa Garcia, ReidyHuang, Yu-ChiehTerrone, GraziaHuerta Ojeda, ÁlvaroTerui, KeitaHuff, AlyssaTesler, RikiHung, Miao-ChiuTesta, GianlucaHursen, CigdemThai, YvonneHurwitz, Lisa B.Thakare, RiteshHussain, Muhammad SajidThapa, PrakashHussain, SaadTheodora, Marianna K.Hussin, Zaharah BintiTheodorakopoulos, AntoniosHusum, Hans-ChristenTheodoridou, StamatiaHwang, SoonjoTheofilis, PanagiotisHwu, Ruth S.Therrell, BradfordIannella, GiannicolaThielemann, FalkIbarluzea, JesusThirunavukkarasu, AshokkumarIbernon, LaureThomaidis, AdinaIbrahim, Mahrous AbdelbassetThomas-Dupont, PabloIbrahim, Maria SalemThompson, Cynthia T.Ide-Okochi, AyakoThompson, George H.Idris, Idayu BadillaThongprayoon, CharatIglesias, AmandaThornberg, Ulrika BirbergIhasz, FerencThumfart, JuliaIhle, AndreasTiefenboeck, ThomasIhn, KyongTierradentro-García, Luis OctavioIkonomidoua, ChrysanthyTilga, HenriIldikó, TarTing, Su-HieIliescu, BogdanTinteren, Harm VanImbery, Terence A.Titterington, Jillİnal, ÖzgüTiwari, AshwiniIndukuri, VijayTiwari, Gyanesh KumarInfante, VictorTodea, DoinaIngelmo, RaquelTodor, Bianca IoanaIngrassia, MassimoTofil, NancyInnocenti, MatteoTogay, EvrinInoue, SachikoTohănean, Dragoș IoanIntra, Francesca SangiulianoTokłowicz, MałgorzataInvernizzi, MarcoToledano-Toledano, FilibertoIrestorm, ElinTolentino-Mayo, LizbethIsaacs, Serena AnnTomás, CatarinaIsac, RalucaTomaszewski, RyszardIshimaru, MihoTomczak, Michał T.Ishimaru, TetsuyaTomé, GinaIskusnykh, Igor Y.Tomlin, Stephen R.Islam, JahidulTompkins, ConnieIslam, Md RabiulTomuschat, ChristianIssing, PeterTonetti, LorenzoIto, TadashiToporowski, GregorIvanišević, AnaTorr, Carrie B.Iwasa, HajimeTorrego Egido, LuisIwata, YoTorres López, María IsabelIyire, AffiongToscano, CarolinaJacinthe, DionTosello, BarthelemyJackowski, TomaszTotan, MariaJackson, Bianca N.Toth, AttilaJacob, SheryTottori, NobuakiJacobs, JohnTouil, NadiaJadhav, RohanTovar-Gálvez, María IsabelJaehne, Anja KathrinTovilla-Zarate, Carlos AlfonsoJalnapurkar, SawanToyoshima, DaisakuJalo, ElliTraficante, DanielaJamilian, AbdolrezaTrager, RobertJan, Sheng-LingTram, NathalieJanczar, SzymonTrambitas, CristianJanjetovic, ZoricaTran, Cheryl L.Jankauskiene, RasaTran, VietJankovic, MomciloTraore, Afsatou NdamaJankowicz-Szymańska, AgnieszkaTrapani, SandraJannat, KanizTregouet, PaulJanuszkiewicz-Lewandowska, DanutaTremolada, MartaJape, GayatriTrentzsch, KatrinJarocka-Cyrta, ElzbietaTriarico, SilviaJaroń, Aleksandra AgnieszkaTripathi, TakshashilaJarosz-Chobot, PrzemysławaTripon, FlorinJasiewicz, BarbaraTrisolino, GiovanniJaskiewicz, LukaszTrosman, IrinaJaskulska, SylwiaTroussas, ChristosJavor, SanjaTrue, LarissaJayaraman, JayakumarTrzcińska, SandraJayaraman, SelvarajTrzcionka, AgataJedrzejczak, WiktorTrzebiński, WojciechJee, Yong-SeokTsai, Han MouJefferies, Craig A.Tsai, Sung Huang LaurentJehangir, SusanTsaknakis, KonstantinosJelski, WojciechTsao, Po-NienJenny, PruefeTsartsalis, Athanasios N.Jensen, OlgaTschauner, SebastianJesus, Luis M. T.Tseilikman, VadimJhe, GraceTsevreni, IridaJiang, RulanTsigkou, VasilikiJiao, BahaiTsitsikas, Dimitris A.Jidovtseff, BorisTsotsi, StellaJiménez Lasserrotte, María del MarTsukahara, HirokazuJiménez, María SaludTucker, Carole A.Jiménez-Gaspar, AmeliaTummolo, AlbinaJiménez-Osorio, Angélica SaraíTurgay, CokyamanJin, WanzhuTurkalj, MirjanaJin, YuefeiTurudić, DanielJing, MengguoTustumi, FranciscoJîtcă, GeorgeTutor, James D.Johnson, BrittanyTwelker, J. DanielJohnson, RebeccaTyler, RichardJohnston, EmilyTyutyusheva, NinaJones, AnnaTzanev, PeterJones, DanU.Raysoni, AmitJones, LouiseUbeda, CarlosJones, RobertUchida, HajimeJooma, ZainubUeda, KaoriJoos, RikUher, IvanJorda, AnselmUjfalusi, AnikóJordan, Anne-KatrinUlici, AlexandruJordan, SusanneUllmann, NicolaJouhar, RizwanUmapathi, Krishna KishoreJovandaric, MiljanaUmemura, AkiraJózsa, GergőUmiastowska, DanutaJuan de Dios, Benítez SilleroUpshur, Carole C.Juan Jesus, Fernandez AlbaUrbanowicz, TomaszJudge, DebraUsán Supervía, PabloJukić, MiroUtesch, TillJump, DeborahUwiera, T. C.Jun, Mi-KyoungVacaroiu, Ileana AdelaJuncar, MihaiVaiopoulou, JulieJunça-Silva, AnaValasoulis, GeorgeJung, Eun HyeValdivia-Moral, PedroJungbauer, FredericValentine, Gregory C.Jungbluth, PascalValenzuela, RodrigoJunge, NormanValero-Chilleron, María JesúsKabesch, MichaelVali, PayamKadiam, Chinna Venkata SubbaiahValladares-Garrido, Mario J.Kaestner, MichaelVallianatos, StephanieKagami, MasayoVallone, FedericaKahl, FritzValtolina, Giovanni GiulioKairys, StevenValtuille, RodolfoKaissi, AliVan De Ven, Cornelis P.Kakar, MohitVan Den Boogaard, Marie-JoseKakavas, SotiriosVan Der Burgt, InekeKalafati, MariaVan Der Linde, Berdien W.Kaliora, Andrianavan Dijk, Jitse P.Kalogiannakis, MichailVan Dokkum, Nienke H.Kalus, AlicjaVan Gemert, Martin J. C.Kamal, Muhammad ShahzadVan Hoorenbeeck, KimKambas, Antonisvan IJzendoorn, SvenKambouri, KaterinaVan Pul, CarolaKamity, RanjithVan Schie, CharlotteKamke, KristynVan Waelvelde, HildeKammar-García, AshuinVande Walle, Johan G. J.Kamogashira, TeruVandenplas, YvanKang, Lin-JuVania, AndreaKaňková, KateřinaVaradharajan, VenkateshwariKanoh, HirokoVarallo, GiorgiaKansal, VikashVarda, NatašaKantor, JiříVardasca, RicardoKantor, Leslie M.Varga, MarcellKao, Chun-ChiehVargas, Jorge H.Kaplan, AlanVarillas-Delgado, DavidKappenberg-Nitescu, Diana CristalaVarlas, Valentin NicolaeKapus, JernejVartholomatos, GeorgeKarakas, CemalVasconcelos, CarlosKaram, Simone M.Vasconcelos-Castro, SofiaKaraman, SaitVasić Anićijević, Dragana D.Karanth, DivakarVasickova, JanaKarapostoli, AimiliaVásquez-Gómez, JaimeKarimli, LeylaVassilakou, ToniaKarlik, JoelleVastyan Md, AttilaKarlović, ZoranVecchia, Stefania DellaKarpava, SviatlanaVeerapandiyan, AravindhanKarthigesu, KandeepanVeerkamp, JaapKasten, ErichVega-Muñoz, AlejandroKatayama, AkihikoVégh, DánielKatzilakis, NikolaosVelasco, Alberto AdaneroKaur, HarpreetVendemmia, MariaKaushik, MaheshVentriglia, FlaviaKawalec, AnnaVerbruggen, SaschaKawamoto, TetsuyaVerd, SergioKazakova, RadaVerlooy, JorisKazanowska, BernardaVerma, Anil K.Kaźmierczak-Wojtaś, NataliaVerma, Vijay KumarKaźmierczyk, JerzyVermeire, KatrienKedem, RonVermeulen, Jeroen R.Keenan, MickeyVermeulen, NathalieKeikha, MasoudVernon-Roberts, AngharadKeil, Margaret F.Vertigan, Anne E.Keil, ThomasVetra, AnitaKeller, JenniferViala, JeromeKelly, CatrionaVidal Carulla, ClaraKelly, DervlaVieira, MargaridaKelly, FionaVijayasekaran, ShyanKemp, KathleenVijlbrief, Daniel C.Kendall, GarthVilchis-Gil, JennyKenđel Jovanović, GordanaVillacis-Nunez, SofiaKenis, VladimirVillanueva, JulioKershner, JohnVillegas Lirola, FranciscoKetter, RalfVilović, MarinoKezić, AnaVinall Miller, JillianKhacharem, AimenVinolo Gil, Maria JesusKhalaf, AtikaVirgone, CalogeroKhaliq, AsifVisscher, Marty O.Khan, HarisVital-López, LourdesKhan, Janine Y.Vitiello, BenedettoKhan, Masood AlamVittinghoff, MariaKhasawneh, FadiVittori, AlessandroKhassawna, Thaqif ElVivalya, Bives Mutume NzanzuKhosravi, Mohammad HosseinVives-Corrons, Joan-LluisKhurshid, MohsinVlasa, AlexandruKhurshid, ZohaibVoicu, CristianaKikuchi, ShogoVolpe, AndreaKim, EugeneVostrý, MichalKim, Hyung-IlVotava-Smith, Jodie K.Kim, Ji YoungVukomanović, Vladislav A.Kim, Jong InVundavalli, SudhakarKim, Ju HeeVyas, PriyankaKim, Jung-WookVymazal, TomasKim, KyungyeolWaak, MichaelaKim, Sang‐DolWada, HideoKim, Seung HwanWada, RebekahKim, Seung YeonWade-Bohleber, Laura MariaKim, Tae ImWadhwaniya, ShirinKim, UshaWagner, Allison F.Kim, Yun JinWagner, LarsKimball, JamesWaitoller, FedericoKimura, YukikoWajchenberg, MarceloKipfmueller, FlorianWajner, SimoneKirby, AnnWakholi, PeterKiss, OrsolyaWald, MartinKitakata, HirokiWalicka-Serzysko, KatarzynaKitamura, TakuroWalker, TimKlaiman, CherylWallen-Russell, ChristopherKlein, Andrés D.Walory, JarosławKlizienė, IrinaWalsh, JuliaKlotz, DanielWalter, Suzy MascaroKnisel, ElkeWang, Hung-HsiangKnitschke, MichaelWang, Liang-JenKo, AraWang, Pei-JungKo, Hsin-chiWang, YueqiKo, Po-JuiWang, ZhengKobicheva, AleksandraWasif, NaveedKoch, KatherineWasilewska, ElizaKoch, Peter J. KochWasnick, RoxanaKocyłowski, R.Wasniewska, Malgorzata GabrielaKodama, YoshihikoWatkeys, Oliver J.Koechlin, HelenWawryk-Gawda, EwelinaKoh, YoungHwanWegscheider, KarlKohen, Viviana LoriaWei, ChangweiKohl, KatharinaWei, LaiKohli, UtkarshWeiner, DanaKoichi, HashimotoWeis, SusanneKojundzic, Sanja LovricWeiss, Jennifer E.Kokić, Ivana BatareloWells, MikeKölbel, HeikeWengrovius, CarissaKolen, Angela M.Weres, AnetaKolodziej, MalgorzataWertzner, Haydèe FiszbeinKolpa, MalgorzataWesemann, WolfgangKoltz, Michael T.Wessel, LucasKomatsu, KeijiWestra, JohannaKomori, KojiWetering, Marianne Desiree Van DeKonbaz, FaisalWhitaker, Amanda T.Kondapelli, Narendra BabuWhite, Anne M.Konduri, Girija GaneshWhite, Ashley A.Konstantinov, VsevolodWhite, HankKopač, MatjažWhiting, SusanKopciuch, DorotaWidasari, Edita RosanaKordek, AgnieszkaWięch, PawełKorinthenberg, RudolfWiechers, CorneliaKornacki, JakubWięckiewicz, MieszkoKorotaeva, EvgeniyaWiener, John S.Korybalska, KatarzynaWiernicka, MarzenaKosami, KokiWilcox, GabrielleKosicka-Gębska, MałgorzataWilczyński, JacekKossakowska, KarolinaWilk, RobertKosti, RenaWilliams, Bethany D.Koszela, KamilWilliams, Ryan A.Koul, Roshan LalWilliams, Sara E.Koumpagioti, DespoinaWilliams, Sian AndreaKoupparis, AndreasWillis, StevenKovacevic, SanjinWilson, CallumKovach, Joshua R.Wilson, Jeffrey M.Kovács, AnitaWilson, RobinKowalewski, GrzegorzWiltshire, Cynthia ArrayaKowalska, Jolanta E.Winings, KathyKozieł, SławomirWirya, StephenKozinc, ŽigaWismayanti, Yanuar FaridaKozioł-Kozakowska, AgnieszkaWisoff, Jeffrey H. WisoffKoźlenia, DawidWohlers, LarsKozlowski, KarlWójcik, MałgorzataKožuh, InesWojsyk – Banaszak, IrenaKranz, StefanWojtowicz, AndreaKrawiec, PaulinaWolak, PrzemyslawKreicbergs, UlrikaWolfsberger, Christina H.Krentel, HaraldWoll, BencieKrieghoff-Henning, EvaWolny, TomaszKriegshauser, GernotWong, AlexanderKrishnan, VijaiWong, Duo Wai ChiKristufkova, ZuzanaWong, Kenneth K. Y.Krogh, Marianne T.Wong, RebeccaKrohne, Tim U.Wong, Yin PingKruger, EstieWongtanasarasin, WachiraKruszewski, ArturWoods, Charles RKuan, GarryWoodward, Kristine ElizabethKubiatko, MilanWouldes, Trecia A.Kubrak, TomaszWozniak, JacekKuchciak, MaciejWren, DouglasKudchadkar, Sapna R.Wright, Geoffrey A.Kühnisch, JanWu, Cheng-LinKui, Andreea IulianaWu, Cheng-TienKuitunen, Ilari M.Wu, Ping-HsunKukić, FilipWu, YangKul, EsraWulczyn, FredKuliczkowski, KazimierzWunderlich, ShahlaKulkarni, DeepaWurtele, Sandy K.Kumagai, NarimasaWyatt, John StephenKumar, AshokWychowański, PiotrKumar, BinodWynne, James H.Kumar, G AjayWyver, ShirleyKumar, RajXaplanteris, PanagiotisKumar, SandeepXhajanka, EditKung, Yen-YingXie, WubinKunz, FelixXu, FengKuo, David C. L.Xu, JinyuKuo, Jinn-RungXu, XiaoheKurath-Koller, StefanXue, HaoKuroki, HiroshiYael, FogelKurtz-Nelson, EvaYagdiran, AylaKurylak, AndrzejYagiela, Lauren M.Kuryłowicz, AlinaYamaguchi, MasaruKvalsvig, JaneYamaguchi, SatoshiKwak, Sang HoYamanouchi, SohsakuKwan Lo, ChungYamatsu, KojiKwan, Christine ManlaiYamauchi, Melissa S.W.Kwiatkowski, StanisławYang, HaiyanKwok, HeatherYang, KunKwon, Jeong-YiYang, NanKwong, AmandaYang, Pei-RungKwong, Wai-HangYang, Wai YewKybartas, Tyler J.Yang, XiaoKyriakidis, IoannisYang, YeonmiKyriakopoulos, MarinosYat Hong Kwan, KennyLa Pergola, EnricoYatsugi, HarukazeLaborie, SophieYawn, BarbaraLachowicz, KatarzynaYe, LiangLadang, AurélieYee, Kimbo E.Ladenhauf, Hannah NoemiYeh, Chien-HsienLaforgia, AlessandraYen, Cheng-ChiehLaGasse, BlytheYen, Cheng-FangLago-Urbano, RocíoYeung, C. AlbertLagunas-Rangel, Francisco AlejandroYi, YangtianLah Tomulić, KristinaYildiz, ErenLakkireddy, Dhanunjaya R.Yilmaz, Berna SekerLaliotis, NikolaosYilmaz, ResulLan, XiaoyuYin, MengLancaster, Hope SparksYip, JoanneLandegger, Lukas D.Yokoi, AkikoLandires, IvanYoon, Sang NamLane, Andrew M.Yorsaeng, RitthideachLane, Shelly J.Youker, RobertLange, EwaYoung, Christopher J.Lange, KarinYoung, JeanineLangiano, ElisaYoung, Philip J.Larrea, LuisYoxall, Charles WilliamLarrosa Haro, AlfredoYu, RondyLarsen, KnudYu, ZhipingLasota, AgnieszkaYum, Yen NaLasota, DorotaYun, YueLatiri, ImedZaami, SimonaLaudańska-Krzemińska, IdaZabala Baños, CarmenLaufer, AndreaZaborowska-Sapeta, KatarzynaLaurenti, PatriziaZabrodskaja, AnastassiaLaurie, KarenZaccarelli-Marino, Maria AngelaLauriti, GiuseppeZadarko-Domaradzka, MariaLawrence, DanaZagalaz-Anula, NoeliaLazarescu, Adrian EmilZaitsu, MasafumiLazarević, DraganaZając, DominikaLázaro, LuisaZakzuk, JosefinaLazzeri, GiacomoZamani, RezaLebaka, EdwardZanchi, PaolaLebel, David EduardZangiacomi Martinez, EdsonLecendreux, MichelZapotoczny, GregLee, Ching-SungZaramella, PatriziaLee, EvelynZare, HosseinLee, Henry C.Zaręba, KorneliaLee, Jae HooZarobell, JohnLee, Jin-HeeZarowski, MarcinLee, MyoungsookZavala-Arciniega, LuisLee, Shih-ChunZavala-Tecuapetla, CeciliaLee, So YoungZdanowicz, KatarzynaLee, YoonJungZdun-Ryżewska, AgataLee, Yuan-TiZdziarski, KrzysztofLehmacher, WalterZechendorf, ElisabethLemajic-Komazec, SlobodankaZhang, Han YuLemos, Gina C.Zhang, XiaoxiaLenártová, PetraZhang, YanjieLengyel, ZsuzsannaZhang, YueyunLentze, MichaelZhao, YunLeon Guerrero, RachaelZhou, HongweiLeonardi, BeatriceZhou, QingLeonhart, RainerZhou, ShengmeiLeón-Rubio, José-MaríaZhou, ZhijunLera, María JoséZia, NukhbaLerma, AbelZielińska, Monika A.Lešták, JánZieliński, GrzegorzLeung, AmyZijlmans, WilcoLevante, AnnalisaZimmer, ChantelleLeventon, JacquelineZimmermann, PeterLevine, Diane ThembekileZimmitti, GiuseppeLevy, Philip ThalerZin, OliviaLewandowski, MichałZingoni, AndreaLewis, Frances MarcusZiółkowska, LidiaLewis, Krystal M.Zisapel, NavaLewis, RuthZissler, UlrichLezine, DeQuincy A.Zlatev, ZlatinLi, Dion Tik ShunZnyk, MałgorzataLi, Hsueh-YuZolotukhin, Igor A.Li, JinglingZuccotti, GianvincenzoLi, PengŻuk, MałgorzataLi, PhilipZumbach, JelenaLi, WeixinZurawski, ArkadiuszLi, YachaoZurc, JocaLiang, ChunZuzda, JolantaLiao, Chien-HungZweifel, PeterLiao, Yu-TsoZwierz, AleksanderLiberska, Hanna



